# Relationships between corneal biomechanics and the structural and functional parameters of glaucoma damage

**DOI:** 10.5935/0004-2749.20200019

**Published:** 2020

**Authors:** Mümin Hocaoğlu, Caner Kara, Emine Malkoç Şen, Faruk Öztürk

**Affiliations:** 1 İstanbul Retina Institute, İstanbul, Turkey; 2 Department of Ophthalmology, Etlik Zübeyde Hanim Women’s Health Education and Research Hospital, Ankara, Turkey; 3 Department of Glaucoma, Ulucanlar Eye Educaton e Research Hospital, Ankara, Turkey; 4 Department of Ophthalmology, Faculty of Medicine, Hacettepe University, Ankara, Turkey

**Keywords:** Corneal pachymetry, Optic disk, Glaucoma, Tonometry, ocular, Nerve fibers, Retina, Visual field, Paquimetria corneana, Disco óptico, Glaucoma, Tonometria ocular, Fibras nervosas, Retina, Campos visuais

## Abstract

**Purpose:**

To investigate the relationships between (i) thickness of the retinal nerve fiber layer, optic nerve head topography, and visual field parameters and (ii) corneal biomechanical properties in normal controls and patients with ocular hypertension and primary open-angle glaucoma.

**Methods:**

This observational, cross-sectional study included 68 eyes with primary open-angle glaucoma, 99 eyes with ocular hypertension and 133 control eyes. Corneal biomechanical properties, optic nerve head topographic features, retinal nerve fiber layer thickness, and visual fields were assessed in all cases. Corneal biomechanical properties, retinal nerve fiber layer thicknesses, and optic nerve head topographic features were compared among the groups. The associations between structural and functional measures of glaucomatous damage and corneal biomechanical factors were also evaluated.

**Results:**

Significantly lower corneal hysteresis and corneal resistance factor values were observed in the primary open-angle glaucoma and ocular hypertension groups as compared with the control group, but there were no significant differences between the primary open-angle glaucoma and ocular hypertension groups. In the ocular hypertension group, no associations were observed between the corneal hysteresis and corneal resistance factor with values and the structural and functional parameters. In the primary open-angle glaucoma group, positive correlations were observed between the corneal hysteresis values and the global retinal nerve fiber layer thickness (p<0.01, r=0.27), mean retinal nerve fiber layer thickness (p<0.01, r=0.33), and mean deviation (p<0.01, r=0.26), and negative correlations were observed between the corneal resistance factor values, and the cup area (p<0.01, r=-0.39), cup-to-disk ratio (p=0.02, r=-0.28), linear cup-to-disk ratio (p=0.02, r=-0.28), and cup shape (p=0.03, r=-0.26). In the control group, weak correlations were detected between the corneal hysteresis and the cup area (p=0.03, r=0.19), cup-to-disk ratio (p=0.01, r=0.21), and linear cup-to-disk ratio (p=0.01, r=0.22).

**Conclusions:**

Distinct correlations were identified between the corneal hysteresis and corneal resistance factor values and the functional and structural parameters in the primary open-angle glaucoma and control groups. Corneal hysteresis and corneal resistance factor may have different roles in the pathophysiology of glaucoma.

## Abstract (pt)

**Objetivo:**

Investigar as relações entre (i) espessura da camada de fibras nervosas da retina, topografia do nervo óptico e parâmetros do campo visual e (ii) propriedades biomecânicas da córnea, em controles normais e pacientes com hipertens ão ocular e glaucoma primário de ângulo aberto.

**Métodos:**

Este estudo observacional, transversal, incluiu 68 olhos com glaucoma primário de ângulo aberto, 99 olhos com hipertensão ocular e 133 olhos controle. As propriedades biomecânicas da córnea, as características topográficas da cabeça do nervo óptico, a espessura da camada de fibras nervosas da retina e os campos visuais foram avaliados em todos os casos. As propriedades biomecânicas da córnea, a espessura da camada de fibras nervosas da retina e as características topográficas da cabeça do nervo óptico foram comparadas entre os grupos. As associações entre medidas estruturais e funcionais de danos glaucomatosos e fatores biomecânicos da córnea também foram avaliadas.

**Resultados:**

Valores de histerese corneana e da resistência corneana foram significativamente menores nos grupos com glaucoma primário de ângulo aberto e hipertensão ocular em com paração ao grupo controle, mas não houve diferenças significativas entre os grupos de glaucoma primário de ângulo aberto e hipertensão ocular. No grupo com hipertensão ocular, não foram observadas associações entre histerese da córnea e o fator de resistência corneana com os valores e os parâmetros estruturais e funcionais. No grupo com glaucoma primário de ângulo aberto foram observadas correlações positivas entre os valores de histerese corneana e a espessura a camada de fibras nervosas da retina (p<0,01, r=0,27), espessura média da camada de fibras nervosas da retina (p<0,01, r=0,33) e desvio médio (p<0,01, r=0,26), e correlações negativas entre o os valores do fator de resistência da córnea e a área de escavação (p<0,01, r=-0,39), a relação escavação/disco (p=0,02, r=-0,28), a relação copo-para-disco linear (p=0,02, r=-0,28) e a forma da escavação (p=0,03, r=-0,26). No grupo controle, correlações foram detectadas entre a histerese da córnea e área de escavação (p=0,03, r=0,19), relação escavação/disco (p=0,01, r=0,21) e relação copo-para-disco linear (p=0,01, r=0,22).

**Conclusões:**

Correlações distintas foram identificadas entre histerese da córnea e os valores de resistência da córnea e os parâmetros funcionais e estruturais nos grupos de glaucoma primário de ângulo aberto e controle. A histerese da córnea e o fator de resistência da córnea podem ter diferentes papéis na fisiopatologia do glaucoma.

## INTRODUCTION

An ocular response analyzer (ORA) is a bidirectional applanation device that is less affected by corneal structure than other devices when estimating corneal biomechanical properties and evaluating intraocular pressure (IOP)^(1)^. With the introduction of ORA, *in vivo* measurements of corneal biomechanical properties, including corneal hysteresis (CH) and the corneal resistance factor (CRF), have become possible for the first time^(2)^. CH reflects both the viscoelastic properties and the “energy absorption capability” of the cornea. CRF is a measure of the total viscoelastic resistance of the cornea to deformation^(1)^.

The determination of the association between corneal biomechanical behaviors and glaucoma remains challenging. Because the cornea, sclera, and lamina cri brosa are contiguous structures, the probable similarities in the biomechanical behaviors of these structures are the main factor supporting this association. According to the mechanical hypothesis of glaucoma, the lamina cribrosa is the main location of damage to the retinal nerve fibers. Wells et al.^(3)^ reported a greater bowing of the optic disk surface in eyes with high CH and IOP. Additionally, a lower CH is observed in patients with glaucoma than in normal subjects, and the disease progresses faster in glaucoma patients presenting with lower CH values^(4-15)^. Studies have reported higher CH values in patients with ocular hypertension (OHT) than in those with glaucoma^(16-19)^.

The relationships between corneal biomechanical properties and glaucomatous structural and functional parameters have also been evaluated in patients with primary open-angle glaucoma (POAG) vs. normal subjects^(20-24)^.

The aim of this study was to determine the relationships between corneal biomechanical properties and the structural and functional measures of glaucomatous damage such as thickness of the retinal nerve fiber layer (RNFL), optic nerve head (ONH) topography, and visual field parameters of patients with OHT and POAG vs. normal patients.

## METHODS

The cohort of this observational, cross-sectional study included patients with POAG and OHT, and a control group of healthy volunteers with no history of systemic or ocular pathology other than refractive errors. The study protocol was approved by the Clinical Research Evaluation Committee of Ankara University, School of Medicine (Ankara, Turkey) (approval no. 14-290) and was conducted in accordance with the tenets of the Declaration of Helsinki. Informed consent was obtained from all participants.

The study group consisted of consecutive patients who were treated for glaucoma and OHT within the pre vious 6 months, and the control group consisted of patients who were treated in outpatient clinics. The desired power level was set to 0.80.

All participants underwent a comprehensive ophthalmological examination, which included medical history, visual acuity, refraction, IOP measurements via ORA and Goldmann applanation tonometry, pachymetric measurements using an ultrasonic pachymeter (UP 1000 Ultrasonic Pachymeter; Nidek Co. Ltd., Tokyo, Japan), gonioscopy using a Goldmann three-mirror lens, and a slit-lamp examination. All patients (not the controls) underwent automated perimetry (Humphrey 750i Visual Field Analyzer; Carl Zeiss Meditec, Inc., Dublin, CA) using the Swedish standard interactive threshold algorithm 24-2. ONH topography was assessed using the Heidelberg Retinal Tomography III confocal scanning laser ophthalmoscope (HRT III; Heidelberg Engineering, Heidelberg, Germany), and peripapillary RNFL thickness was assessed using spectral domain optical coherence tomography (Spectralis; Heidelberg Engineering).

The inclusion criteria for patients with POAG were pretreatment IOP>21 mmHg, glaucomatous disk changes, and typical glaucomatous field defects on at least two reliable perimetry tests with an open iridocorneal angle. The inclusion criteria for patients with OHT were IOP >21 mmHg (pretreatment or without treatment) and the absence of optic disc damage with normal visual field and spectral domain optical coherence tomography findings. The inclusion criteria for control subjects were no ocular pathology other than refractive errors and IOP ≤21 mmHg.

Patients with a history of any ocular surgery, trauma, uveitis, pigment dispersion syndrome, pseudoexfoliation syndrome, or secondary glaucoma were excluded from analysis. Patients with systemic conditions that could affect ocular biomechanics (i.e., connective tissue diseases, muscular dystrophies, and thyroid dysfunction) were also excluded, whereas those with diabetes mellitus (DM) were not.

Four ORA measurements were obtained. If both eyes of a participant met the inclusion criteria, the eye with a more reliable ORA measurement and the best waveform score was selected for analysis. All subjects had a waveform score >5.0. The Goldmann-correlated IOP (IOPg), corneal-compensated IOP (IOPcc), CH, and CRF values with the best-quality signal wave were included for statistical analysis. ORA was used to measure four variables: CH, CRF, IOPcc, and IOPg. The device was also used to measure the air pressure required to flatten the cornea. Two independent applanation pressures were applied for inward and outward corneal deformation. Owing to the corneal biomechanical properties, the first applanation pressure was greater than the second. The difference between the two pressures is defined as CH, which is believed to indicate the viscoelastic properties of the cornea^(2)^. CH indicates corneal viscous damping, which is the ability of corneal tissue to absorb energy. CRF is a variable derived from CH and is a linear combination of applanation pressures, indicating overall corneal resistance, which correlates with the central corneal thickness (CCT)^(25)^. IOPcc is an IOP measurement calculated from CH data and is suggested to be less affected by the corneal structure^(2)^. IOPg is the average of the two applanation pressures.

Statistical analyses were performed using IBM SPSS Statistics for Windows, version 20.0 (IBM Corporation, Armonk, NY). Categorical variables were compared using the chi-square test. One-way analysis of covariance was used to compare the groups after adjusting for the confounding factors of IOP, CCT, age, axial length, and DM. The Bonferroni post hoc test was used for pairwise comparisons of CH and CRF between the diagnostic groups. Multiple linear regression analyses were used to evaluate the relationships between the CH and CRF values and the visual field, nerve fiber thickness, and HRT parameters after adjusting for potential confounders. Potential confounders for the analysis of relationships between the CH and CRF values were disc area, IOP, CCT, age, axial length, and OHT treatment status. Potential confounders to assess the relationship between the CH and CRF values and the visual field and nerve fiber thickness included HRT parameters, IOP, CCT, age, axial length, and OHT treatment status. A probability (p) value of <0.05 was considered to be statistically significant.

## RESULTS

Data from 68 eyes in the POAG group, 99 eyes in the OHT group, and 133 eyes in the control group were analyzed. The demographic and clinical characteristics of the groups are summarized in table 1.

**Table 1:** Demographic and clinical characteristics of patient and control groups

	Control		OHT	POAG	*P*
Number	n (%)	133 (44.7%)	99 (32.8%)	68 (22.5%)	-
Sex	Male (n, %)	64 (52.6%)	40 (59.6%)	34 (50.0%)	n/s
	Female (n, %)	69 (47.4%)	59 (40.4%)	34 (50.0%)	
DM	n (%)	30 (22.6%)	10(10.1%)^*^	14 (20.6%)	<0.001
Age (years)	Mean ± SD	55.43 ± 8.65	56.71 ± 8.63	62.96 ± 8.15^*^	<0.001
	(Range)	(41-77)	(40-79)	***(51-80)***	
IOP (mmHg)	Mean ± SD	*15.73* ± 2.94^*^	18.34 ± 4.66	16.65 ± 5.42	<0.001
	(Range)	(10.00-20.00)	(10.00-30.00)	(10.00-28.00)	
CCT (µm)	Mean ± SD	543.69 ± 28.19	559.16 ± 37.01^*^	539.54 ± 33.37	<0.001
	(Range)	(470-605)	***(481-645)***	(477-617)	
Axial length (mm)	Mean ± SD	23.07 ± 0.87	23.10 ± 0.53	23.12 ± 0.93	n/s
	(Range)	(20.50-24.99)	(22.19-24.62)	(21.10-25.51)	
Number of medications	Mean ± SD	-	0.87 ± 0.53	1.24 ± 0.43^*^	<0.001
	(Range)	-	(0.00-2.00)	***(1.00-2.00)***	

CCT= central corneal thickness; DM= diabetes mellitus; IOP= intraocular pressure; n/s= not significant; OHT= ocular hypertension; POAG= primary open-angle glaucoma; SD= standard deviation.

* significant at *p*<0.001.

**Figure 1:**
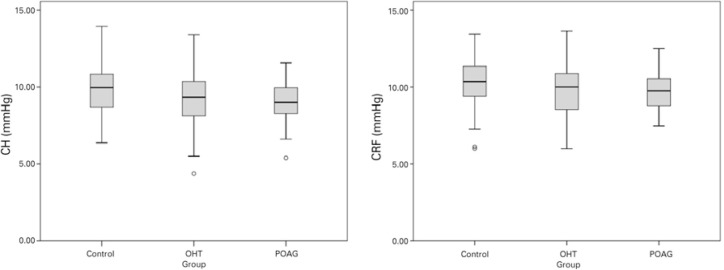
Boxplot representation of CH and CRF values of each group with 95% confidence intervals.

By crude analysis without adjusting for CCT, age, IOP, axial length, or the presence of DM, the CH and CRF values were higher in the OHT and control groups than in the POAG group, and there were no significant differences between the OHT and control groups. After adjusting for these confounders, the CH and CRF values were significantly lower in the POAG and OHT groups than in the control group, and there were no significant differences between the POAG and OHT groups. By subgroup analysis of the OHT group according to treatment status, no significant differences were observed in the CH and CRF values between OHT eyes with vs. without treatment (p=0.99 and 0.66, respectively). The corneal biomechanical properties and the boxplot representation of the three groups are presented in table 2 and figure 1, respectively.

**Table 2:** Corneal biomechanical properties of the groups

		Control	OHT	POAG	p
CH (mmHg)	Mean ± SD	9.88 ± 1.51^*^	9.38 ± 1.95	8.74 ± 1.46	<0.05
	(Range)	**(6.40-14.30)**	(4.60-13.10)	(4.10-11.60)	
CRF (mmHg)	Mean ± SD	10.07 ± 1.75^*^	10.37 ± 2.31	9.46 ± 1.96	<0.05
	(Range)	**(5.10-15.20)**	(5.40-15.10)	(6.40-14.50)	
IOPcc (mmHg)	Mean ± SD	16.97 ± 3.63^**^	20.07 ± 5.29	18.76 ± 6.32	<0.001
	(Range)	**(9.50-23.70)**	(9.90-41.40)	10.90-41.40)	
IOPg (mmHg)	Mean ± SD	15.92 ± 3.86^**^	18.8 ± 5.6	17.01 ± 6.86	<0.001
	(Range)	**(7.00-25.10)**	(7.90-38.90)	(7.80-38.90)	

CH= corneal hysteresis; CRF= corneal resistance factor; IOPcc= corneal-compensated IOP; IOPg= Goldmann-correlated IOP; OHT= ocular hypertension; POAG= primary open-angle glaucoma; SD= standard deviation.

* significant at *p*<0.05;

** significant at *p*<0.001.

In terms of the structural and functional measures of glaucomatous damage, the POAG group had significantly worse values than the other two groups. The structural and functional measures of glaucomatous damage of each group are presented in table 3.

**Table 3:** Structural and functional parameters of patient and control groups

		Control	OHT	POAG	p
Mean deviation (dB)	Mean ± SD	-	1.38 ± 1.35	-9.55 ± 3.50^*^	<0.001
	(Range)		(-4.15 to 3.03)	**(-18.28 to -3.86)**	
Pattern standard deviation (dB)	Mean ± SD	-	1.92 ± 0.52	7.80 ± 2.02^*^	<0.001
	(Range)		(1.00-3.00)	**(4.33**-**12.47)**	
RNFL thickness (µm)	Mean ± SD	100.50 ± 9.36	97.35 ± 11.31	80.71 ± 17.66^*^	<0.001
	(Range)	(79.00-122.00)	(52.00-123.00)	**(39.00**-**114.00)**	
Disk area (mm^2^)	Mean ± SD	2.29 ± 0.44	2.20 ± 0.43^*^	2.44 ± 0.45	<0.001
	(Range)	(1.00-3.50)	**(1.00**-**3.60)**	(1.59-3.62)	
Cup area (mm^2^)	Mean ± SD	0.49 ± 0.35	0.50 ± 0.29	1.22 ± 0.41^*^	<0.001
	(Range)	(0.00-1.63)	(0.00-1.30)	**(0.55**-**2.25)**	
Rim area (mm^2^)	Mean ± SD	1.81 ± 0.45	1.69 ± 0.32	1.21 ± 0.34^*^	<0.001
	(Range)	(0.00-4.43)	(1.00-2.54)	**(0.27**-**2.15)**	
Cup volume (mm^3^)	Mean ± SD	0.12 ± 0.14	0.12 ± 0.11	0.45 ± 0.28^*^	<0.001
	(Range)	(0.00-0.93)	(0.00-0.58)	**(0.06**-**1.45)**	
Rim volume (mm^3^)	Mean ± SD	0.50 ± 0.20	0.47 ± 0.16	0.29 ± 0.21^*^	<0.001
	(Range)	(0.20-1.56)	(0.17-1.07)	**(0.03**-**1.60)**	
CDR	Mean ± SD	0.20 ± 0.11	0.22 ± 0.10	0.50 ± 0.10^*^	p<0.001
	(Range)	(0.01-0.39)	(0.01-0.40)	**(0.41**-**0.83)**	
Linear CDR	Mean ± SD	0.42 ± 0.14	0.45 ± 0.13	0.70 ± 0.07^*^	<0.001
	(Range)	(0.04-0.62)	(0.10-0.63)	**(0.58**-**0.91)**	
Mean cup depth (mm)	Mean ± SD	0.21 ± 0.13	0.21 ± 0.09	0.36 ± 0.10^*^	<0.001
	(Range)	(0.01-1.17)	(0.05-0.56)	**(0.11**-**0.56)**	
Maximum cup depth (mm)	Mean ± SD	0.56 ± 0.25	0.60 ± 0.22	0.81 ± 0.20^*^	<0.001
	(Range)	(0.02-1.32)	(0.16-1.34)	**(0.21**-1.39)^*^	
Cup shape	Mean ± SD	0.20 ± 0.06	0.21 ± 0.06	0.08 ± 0.07^*^	<0.01
	(Range)	(-0.42 to -0.08)	(-0.34 to -0.04)	**(0.25**-**0.08)**	
Mean RNFL thickness (mm)	Mean ± SD	0.26 ± 0.07	0.25 ± 0.07	0.22 ± 0.07^*^	<0.001
	(Range)	(0.10-0.48)	(0.10-0.42)	**(0.01**-**0.41)**	

CDR= cup-to-disk ratio; OHT= ocular hypertension; POAG= primary open-angle glaucoma; RNFL= retinal nerve fiber layer; SD= standard deviation.

* significant at *p*<0.001.

Regarding the associations between visual field parameters and corneal biomechanics, in the OHT group, no correlations were detected between the mean deviation (MD) and pattern standard deviation (PSD) values or between the CH and CRF values (p>0.05). In the POAG group, a weak positive correlation was identified between the MD and CH values (*p*<0.01, r=0.26) and the MD and CRF values (p=0.03, r=0.27). However, there were no significant correlations among the PSD, CH, and CRF values (p>0.05).

No significant correlations were found between the RNFL thickness and the CH and CRF values in the OHT and control groups (p>0.05). In the POAG group, a weak positive correlation was detected between the mean RNFL thickness and the CH values (p<0.01, r=0.27), but not between the mean RNFL thickness and the CRF values (p>0.05).

**Figure 2:**
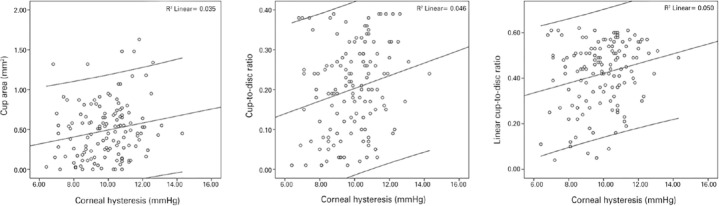
Scatter plots of the observed significant relationships between corneal biomechanics and functional and structural properties in the control group.

**Figure 3:**
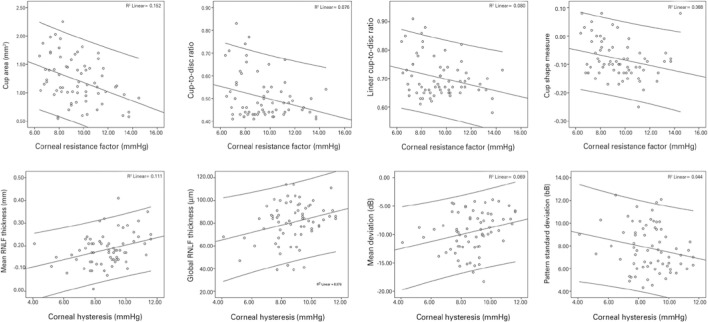
Scatter plots of the observed significant relationships between corneal biomechanics and functional and structural properties in the POAG group.

In terms of associations between structural features of the ONH and corneal biomechanics, in the OHT group, no significant correlations were detected between the CH and CRF values. In the control group, significant correlations between CH and cup area (p=0.03, r=0.19), CDR (p=0.01, r=0.21), and linear CDR (p=0.01, r=0.22) were detected. In the POAG group, weak negative correlations between CRF and cup area (p<0.01, r=-0.39), CDR (p=0.02, r=-0.28), linear CDR (p=0.02, r=-0.28), and cup shape (p=0.03, r=0.26) were detected. Additionally, in the POAG group, a weak positive correlation was detected between the CH value and the mean RNFL thickness (p<0.01, r=0.33). The associations between the CH and CRF values and the structural and functional measures of glaucomatous damage are presented in table 4 and figures 2 and 3.

**Table 4:** Multiple linear regression analysis of the associations between corneal biomechanics and structural and functional measures of glaucomatous damage

	Control						OHT						POAG				
	CH				CRF		CH			CRF			CH			CRF	
	B	SE(B)	β	B	SE(B)	β	B	SE(B)	β	B	SE(B)	β	B	SE(B)	β B	SE(B)	β
Disk area (mm^2^)	-0.01	0.03	-0.02	-0.01	0.03	-0.04	-0.03	0.02	-0.13	-0.01	0.03	-0.06	-0.02	0.04	-0.08 0.03	0.03	0.13
Cup area (mm^2^)	**0.04**	**0.02**	0.16^*^	0.02	0.02	0.09	0.00	0.01	0.02	0.00	0.01	0.00	-0.03	0.03	-0.13 **-0.07**	**0.03**	-0.33^*^
Rim area (mm^2^)	-0.02	0.02	-0.06	0.00	0.02	0.02	0.00	0.01	0.02	0.01	0.02	0.06	0.02	0.03	0.10 0.07	0.03	0.38
Cup volume (mm^3^)	-0.02	0.02	-0.06	-0.01	0.01	-0.10	0.00	0.01	-0.03	-0.01	0.01	-0.15	-0.03	0.03	-0.16 -0.04	0.03	-0.31
Rim volume (mm^3^)	0.00	0.01	-0.02	0.01	0.01	0.05	0.00	0.01	-0.04	0.01	0.01	0.10	0.03	0.02	0.22 0.04	0.02	0.33
Cup-to-disk area ratio	**0.01**	**0.01**	0.17^*^	0.01	0.01	0.15	0.00	0.01	0.06	0.00	0.01	0.07	-0.01	0.01	-0.15 **-0.02**	**0.01**	-0.47^*^
Linear cup-to-disk area ratio	**0.02**	**0.01**	0.19^*^	0.02	0.01	0.18	0.00	0.01	0.07	0.01	0.01	0.11	-0.01	0.01	-0.12 **-0.02**	**0.01**	-0.44^*^
Mean cup depth (mm)	0.01	0.01	0.12	0.01	0.01	0.12	0.00	0.01	0.00	0.00	0.01	0.01	-0.01	0.01	-0.15 -0.01	0.01	-0.10
Maximum cup depth (mm)	0.01	0.01	0.08	0.01	0.02	0.05	0.01	0.01	0.06	0.01	0.01	0.08	0.01	0.02	0.06 0.02	0.02	0.23
Cup shape	0.00	0.00	0.06	0.00	0.00	0.11	0.00	0.00	-0.03	0.00	0.00	0.04	0.00	0.01	-0.08 **-0.02**	**0.01**	-0.53^*^
Mean RNFL thickness (mm)	0.00	0.00	0.02	0.01	0.00	0.12	0.00	0.00	-0.03	0.00	0.01	0.08	0.02	0.01	0.30^*^ **0.01**	**0.01**	0.38^*^
Mean deviation (dB)	n/a	n/a	n/a	n/a	n/a	n/a	0.00	0.07	0.00	0.15	0.08	0.28	**0.83**	**0.35**	0.35^*^ 1.17	**0.31**	0.65^**^
Pattern standard deviation (dB)	n/a	n/a	n/a	n/a	n/a	n/a	0.04	0.03	0.12	-0.01	0.04	-0.03	-0.23	0.20	-0.17 -0.31	0.19	-0.30
RNFL thickness (µm)	0.58	0.53	0.09	0.60	0.55	0.11	-0.35	0.65	-0.06	-0.81	0.73	-0.17	**4.77**	**1.80**	0.40^*^ 4670	1713	0.52

β= standardized beta; B= unstandardized beta; CDR= cup-to-disk ratio; CCT= central corneal thickness; CH= corneal hysteresis; CRF= corneal resistance factor; IOP= intraocular pressure; n/a= not available; OHT= ocular hypertension; POAG= primary open-angle glaucoma; RNFL= retinal nerve fiber layer; SE= standard error for unstandardized beta.

* significant at *p*<0.05;

** significant at *p*<0.001.

## DISCUSSION

In numerous studies, a lower CH value was consistently observed in patients with glaucoma as compared with normal subjects, and disease progressed faster in glaucoma patients with lower CH values. Abitbol et al.^(4)^ reported a CH value of 10.46 ± 1.6 mmHg in the control group (mean age, 61.44 years) and a significantly lower CH value of 8.77 ± 1.4 mmHg in the glaucoma group (mean age, 65.68 years). In a study by Mangouritsas et al.^(13)^, there were significant differences in the CH values between the control (mean age, 59.2 ± 14.2 years) and the glaucoma (mean age, 62.4 ± 9.8 years) groups (10.97 ± 1.59 vs. 8.95 ± 1.27 mmHg, respectively). According to Bochmann et al.^(26)^, patients with an acquired pit of the optic nerve have significantly lower CH values than those without this structural change. Moreover, a prospective study by Susanna et al.^(27)^ reported an association between CH and an increased risk of developing glaucoma. The lower CH values observed in the glaucoma group in the present study confirm the findings of the aforementioned studies.

In studies that included both the OHT and POAG groups, higher CH and CRF values were observed in the OHT group than in the glaucoma group, and there were no significant differences as compared with the healthy control group^(16-19)^. However, the results of the present study do not support this finding. Patients with OHT are a specific group in terms of IOP and CCT, which may affect the dynamic nature of the CH and CRF values^(28)^. The unadjusted analysis of these factors in the present study showed significantly higher CH and CRF values in the OHT group than in the glaucoma group. However, after adjusting for confounding factors that potentially affect the CH and CRF values, no differences were observed. As a possible explanation for this finding, regardless of the number of confounding factors (such as IOP, age, and CCT), some patients in the OHT group were receiving treatment. Nevertheless, a similar result was obtained for patients in the OHT group who were not receiving treatment. However, the patients who rec eived treatment comprised only a small portion of the OHT group (n=21/99). Furthermore, the number of patients in the OHT group (n=99) in the present study was noticeably greater than in previous studies^(16-19)^. An appropriate analysis with a larger sample of OHT eyes without treatment may resolve this discrepancy and confirm these findings.

The relationships between corneal biomechanical properties and glaucomatous structural and functional parameters have been evaluated in various studies, particularly in patients with POAG vs. normal subjects^(20-24)^. For instance, Prata et al.^(23)^ evaluated patients with POAG at diagnosis and identified negative correlations between CH and the CDR and mean cup depth. Moreover, Khawaja et al.^(22)^ identified a positive correlation between CH and rim area and a negative correlation between the linear CDRs in a large-scale population-based study. In contrast, a population-based study by Carbonaro et al.^(24)^ found no significant relationship between CH and the optic disc parameters. In the present study, a relationship was observed between the optic disc parameters and the CRF values in the glaucoma group and between the optic disc parameters and the CH values in the control group.

In a study evaluating the correlation between CH and RNFL thickness, Mansouri et al.^(20)^ found no significant correlation between these parameters in glaucomatous eyes. Vu et al.^(21)^ reported similar results. A study by Khawaja et al.^(22)^ showed a positive correlation between CH and RNFL thickness. Likewise, there was a positive correlation between RNFL thickness and CH in the present study, which supported the findings reported by Khawaja et al.

When the corneal biomechanical features and visual fields were evaluated, De Moraes et al.^(29)^ and Congdon et al.^(30)^ also a correlation between low CH and progressive worsening of the visual field. Mansouri et al.^(20)^ identified positive correlations between the CH and CRF values and the MD and PSD values. However, after adjusting for the confounding factors of CCT, age, and axial length, the positive correlation with CRF remained. In the present study, the correlation between the CH and MD values in the glaucoma group is consistent with the findings of the aforementioned studies.

Interestingly, correlations between the CRF, but not CH, and the structural features of the optic disc were observed in glaucoma patients in the present study. Additionally, prominent relationships between CH, RNFL thickness, and visual field parameters were observed in the glaucoma group. The relationships between CRF and structural features of the optic disc are intriguing.

Although CRF is a parameter of the elastic features of the cornea and assigns more weight to the first applanation pressure assessed during the ORA measurement, higher CRF values require more pressure for the initial corneal applanation. This situation may correspond to a requirement for higher pressures for lamellar deformation that cause axonal damage to the optic disks of individuals with higher CRF values when evaluated at the optic disk level. Therefore, in individuals with higher CRF values, a stronger axonal structure may prevent deformation up to a certain pressure. If the critical pressure level is exceeded, the protective damping properties associated with CH might be activated, and the adverse effects of the pressure on the nerves undergoing deformation may be reduced by damping to prevent axonal damage.

As a result, the greater cup areas, CDRs, and linear CDRs in patients with glaucoma presenting with low CRF values suggest that pressure-induced optical disk deformation is more likely to develop due to the negative relationships between the functional parameters and CH, which reduces the protective effect of CH during damage.

Another interesting finding of our study is the positive correlations between CH values and cup areas, CDRs, and linear CDRs in normal individuals. These relationships between optic disk parameters were not ge nerally observed in previous studies of normal populations^(24,31)^. Although this result appears to contradict the biomechanical properties associated with CH, it may be due to nonpathological cupping formation caused by bowing that occurs even at normal IOP levels because of the high CH value of the control group. The topographic structure of ONH is not static and may even show changes in normal individuals due to the forward and backward movements of the lamina cribrosa in response to intraocular and cerebrospinal pressures^(32)^. Azuara-Blanco et al.^(33)^ evaluated the topographic changes in the optic disk after acute elevation of IOP by HRT and observed increased cupping of the optic disk. In this regard, our finding was similar to the results reported by Wells et al.^(3)^ and may indicate that this phenomenon, which is normally observed at high pressures, can be observed even at normal IOP in individuals with high CH values. The evaluation of CH in patients with physiological cupping may also be useful to clarify this interesting finding.

The main implication of this study for clinical practice is that CH and CRF might have different roles in the pathophysiology of glaucoma. Reduced elasticity at the laminal level associated with low CRF values and protective damping properties associated with high CH values may be taken into consideration when determining the target IOP for glaucoma management^(34)^.

There were some limitations in this study. First, the associations in this study were weak; thus, the clinical relevance of these associations needs to be determined. Second, patients with OHT were not a homogeneous group because those receiving treatment were also included. Third, because the MD and PSD values may vary in normal individuals, the absence of visual field values in the control group may limit the clinical implication of the associations detected in the study. Fourth, by including eyes with the most reliable ORA measurements, randomization was disrupted, which may have influenced the findings.

In conclusion, this study assessed the relationships between corneal biomechanical properties and the functional and structural measures of glaucomatous damage, and identified some interesting correlations, some for the first time. Nonetheless, prospective longitudinal studies are required to confirm these results.
